# Clinical Genetics of Cancer 2017: Szczecin, Poland. 21-22 September 2017

**DOI:** 10.1186/s13053-018-0087-z

**Published:** 2018-02-28

## A1 Genetics of familial cancer

### Hemminki K^1,2^, Försti A^1,2^

#### ^1^Division of Molecular Genetic Epidemiology, German Cancer Research Center (DKFZ), 69120 Heidelberg, Germany; ^2^Center for Primary Health Care Research, Lund University, Malmö, Sweden

The accumulating data on germline variation in various cancers are able to explain between 15 and 50% of the known familial risks. The genetic data derive essentially from 3 different sources, family studies which identified the majority of the known high-risk genes (over 110 genes), genome-wide association study (GWAS) detected genes/loci (close to 400) and finally the analysis of GWAS data for familial clustering for heritability estimates. The proportion of the 3 sources of data contributing to various cancers differs greatly. The germline architecture of breast and ovarian cancer has a major contribution from the high-risk genes while for prostate and lung cancer the major contribution is from low-risk genes. Even though the application of next-generation sequencing (NGS) has hugely increased the number of detected somatic mutations in human cancers no boost in the number of new cancer predisposing genes has been evident. Yet NGS does afford an advantageous technology also for germline sequencing. We believe that the reasons for the lack of success are manifold, including lack of appropriate families with extensive pedigrees, shortcomings in bioinformatics approaches, and, perhaps, wrong expectations about the germline architecture of human cancer. In this presentation we will compare germline approaches between GWAS and NGS technologies and describe some extensions to the existing germline genetic pipelines.

References

Li N, Johnson DC, Weinhold N, Studd JB, Orlando G, Mirabella F, Mitchell JS, Meissner T, Kaiser M, Goldschmidt H, Hemminki K, Morgan GJ, Houlston RS. Multiple myeloma risk variant at 7p15.3 creates an IRF4-binding site and interferes with CDCA7L expression. Nat Commun. 2016;7:13656.

Försti A, Kumar A, Paramasivam N, Schlesner M, Catalano C, Dymerska D, Lubinski J, Eils R, Hemminki K. Pedigree based DNA sequencing pipeline for germline genomes of cancer families. Hered Cancer Clin Pract. 2016;14:16

## A2 Inherited mutations of PALB2 gene and breast cancer

### Cybulski C^1^, Kluźniak W^1^, Huzarski T^1^, Wokołorczyk D^1^, Kashyap A^1^, Rusak B^1^, Jakubowska A^1^, Szwiec M^2^, Byrski T^1^, Dębniak T^1^, Górski B^1^, Sopik V^3^, Akbari MR^3^, Sun P^3^, Gronwald J^1^, Narod SA^3^, Lubiński J^1^, Polish Hereditary Breast Cancer Consortium

#### ^1^Department of Genetics and Pathology, Pomeranian Medical University, Szczecin, Poland. ^2^Regional Oncology Center, Opole, Poland; ^3^Women’s College Research Institute, Toronto, ON, Canada

Mutations in PALB2 predispose to breast cancer. There are two founder mutations of PALB2 in the Polish population (509_510delGA and 172_175delTTGT) which are associated with 4 to 5 –fold increased risk of breast cancer. We found that 10-year survival for women with breast cancer and a PALB2 mutation is worse than of patients with breast cancer without a mutation (48% vs 75%, adjusted HR = 2.3; p<0·0001). Given that women with a PALB2 mutation face a high risk of breast cancer and are at a higher risk of death, increased surveillance should be offered to PALB2 carriers. It should be established whether unaffected PALB2 carriers benefit from prophylactic mastectomy, and if PABL2 carriers with breast cancer benefit from specific treatment, in particular specific chemotherapy regimens.

## A3 Multi-cancer gene panel testing in 1255 Dutch familial cancer patients: single nucleotide and copy number variant analysis

### Sijmons R

#### Department of Genetics, University of Groningen, University Medical Center Groningen, Groningen, The Netherlands

Next Generation Sequencing (NGS) allows diagnostic testing of many genes simultaneously and the use of NGS gene panels is now common in familial cancer diagnostics. In diagnostics, these panels usually target particular tumour types or combination of tumour types, e.g. colorectal cancer, or breast and ovarian cancer, deliberately limiting the chance of unsolicited findings. However, the systematic use in familial diagnostics of broader, multi-cancer gene panels, including new candidate cancer predisposing genes, could potentially help identify expansions of the currently known tumour syndrome phenotypes and could help define the phenotypes of mutations in newly postulated genes. This might, in time, increase the diagnostic yield in patients as most of them are currently left without a molecular diagnosis. Another reason for using broader gene panels is that it would allow for screening of actionable mutations in cancer predisposing genes as has for example been advocated by the American College of Medical Genetics and Genomics. Although such screening, as opposed to diagnostics, is currently not part of Dutch genetic counselling and testing services, the potential benefits of such screening warrant further study. With all these considerations in mind, we developed an 85-gene multi-cancer NGS panel including well-known as well as, for research only, some newly postulated tumour syndrome genes, and implemented it in our diagnostics lab. For diagnostic purposes, clinicians can order reports on particular cancer specific subsets of the panel data, whereas for research purposes, all panel genes can be analysed anonymously in all patients. We analysed the yield of variants in 1,255 patients referred to our clinic for familial cancer diagnostics, and to try to match those variants with the tumour types that had prompted referral for testing. Those that would not match could be regarded as secondary findings and/or as suggestions for future expansions of the known tumour gene phenotypes. In analysing our NGS panel data we aimed to analyse all 85 panel genes for CNVs as well, which is not yet common diagnostic practice. We were interested to know how much CNV analysis in the NGS data for all genes, rather than of selected ones using the traditional Multiplex Ligation-dependent Probe Amplification (MLPA), would add to the single nucleotide variant (SNV) analysis in terms of mutational yield. In order to get an estimate of what to expect in frequency of secondary findings, we analysed both SNV and CNVs in the dataset of all 498 non-related individuals from the Dutch population whole genome sequencing project GoNL. Results will be presented at the meeting.

## A4 Results of cis-platinum treatment of breast cancer patients with brca1 mutation

### Narod S^1^, Byrski T^2^, Marczyk E^3^, Wiśniowski R^4^, Szwiec T^5^, Huzarski T^6^, Cybulski C^6^, Dębniak T^6^, Gronwald J^6^, Lubiński J^6^

#### ^1^Women’s College Hospital, University of Toronto, Toronto, Canada; ^2^Department of Oncology, Pomeranian Medical University, Szczecin, Poland; ^3^Department of Surgical Oncology, Oncology Center, Kraków, Poland; ^4^Department of Oncology, Oncology Center, Bielsko-Biała, Poland; ^5^Department of Oncology, Oncology Center, Opole, Poland; ^6^International Hereditary Cancer Center, Department of Genetics and Pathology, Pomeranian Medical University, Szczecin, Poland

The aim of this study is to evaluate the effectiveness of neoadjuvant treatment with cisplatin chemotherapy in women with breast cancer and a BRCA1 mutation. One 131 women with breast cancer and a BRCA1 mutation, who were diagnosed with stage I to III breast cancer between December 2006 and June 2017, were treated with cisplatin 75 mg/m(2) every 3 weeks for four cycles, followed by mastectomy and conventional chemotherapy. Information was collected on clinical stage, grade, hormone receptor status, and Her2neu status prior to treatment. pCR was determined by review of surgical specimens. Medium time of observation was 52 months. One hundred 131 patients were enrolled in the study. A pCR was observed in 79 (60 %) patients and pPR in 46 (35%). Between 79 patients who obtained pCR 1 patient died.

Platinum-based chemotherapy is effective in a high proportion of patients with BRCA1-associated breast cancer.

## A5 Relatively high incidence of non-founder brca1/2 mutation carriers among familial breast cancer cases in Latvia

### Irmejs A, Maksimenko J, Trofimovičs G, Bērziņa D, Skuja E, Purkalne G, Miklaševičs E, Gardovskis J

#### Oncology Institute, Riga Stradins University, Riga, Latvia

**Background**

In Latvia, pathogenic BRCA1 founder variants contribute to about 3.77% of all consecutive primary breast cancers and about 9.9% of all consecutive primary ovarian cancers. Identifying germline pathogenic gene variants in patients with primary breast and ovarian cancer could significantly impact the patients’ medical management.

**Methods**

10 female and 1 male patients were included. 9 probands had breast cancer, 1 – ovarian and 1 breast and ovarian cancer. In 26 gene panel following genes were included - ATM, BARD1, BLM, BRCA1, BRCA2, BRIP1, CDH1, CHEK2, EPCAM, FAM175A, MEN1, MLH1, MRE11A, MSH2, MSH6, MUTYH, NBN, PALB2, PMS2, PTEN, RAD50, RAD51C, RAD51D, STK11, TP53, XRCC2. All patients were tested negative for the pathogenic variants of the BRCA1 with founder effect (c.181T>G, c.4035delA, c.5266dupC).

**Results**

Of the 11 patients tested, pathogenic variants were identified in 7 (64%) patients: 6 patients carried pathogenic variants of the BRCA1 gene and one of the BRCA2 gene. In 3 patients a variants of uncertain significance of the BRCA2, RAD50 and MRE11A genes was found.

**Conclusion**

A 26-gene panel second-line testing increased the number of positive test results in unsolved primary breast and ovarian cancer patients matching criteria for BRCA1/2 testing.

## A6 Do recurrent mutations in genes other than brca1/2, chek2 and palb2 play important role in predisposition to breast cancer in polish women?

### Kluźniak W^1^, Wokołorczyk W^1^, Rusak B^1^, Kashyap A^1^, Lubiński J^1^, Cybulski C^1,2^

#### ^1^Department of Genetics and Pathology, Pomeranian Medical University, Szczecin, Poland; ^2^Centre for Modern Interdisciplinary Technologies, Nicolaus Copernicus University, Torun, Poland

Approximately 20 genes other than BRCA1, BRCA2, CHEK2 and PALB2 have been associated with breast cancer predisposition, and extended genetic testing panels have been proposed. In Poland we found a number of founder mutations in BRCA1, CHEK2 and PALB2, however it is unknown if founder mutations in other candidate susceptibility genes play an important role in breast cancer in Polish women. Here we sought to establish if a single truncating mutation of XRCC2 (c.96delT, p.Phe32fs) detected in by whole-exome sequencing of 144 Polish women with familial breast cancer is recurrent mutation in Poland and whether it is associated with a genetic susceptibility to breast cancer in the population. We genotyped 3000 women with breast cancer and 2000 heathy women for the c.96delT mutation. The mutation was present with similar frequency in cases (0.23%) and in controls (0.25%). Our data suggest that c.96delT truncating mutation of XRCC2 is not associated with increased risk of breast cancer. This data calls into question adding of XRCC2 gene to breast cancer testing panels.

## A7 Missense mutations of NBS1 and the risk of breast and prostate cancers

### Rusak B^1^, Kluźniak W^1^, Wokołorczyk D^1^, Lubiński J^1^, Cybulski C^1,2^

#### ^1^Department of Genetics and Pathology, Pomeranian Medical University, Szczecin, Poland; ^2^Centre for Modern Interdisciplinary Technologies, Nicolaus Copernicus University, Torun, Poland

The DNA damage signaling pathway plays a crucial role in the maintenance of the integrity of the genome in response to DNA damage and has been implicated in the pathogenesis of prostate cancer. Individuals with an inherited recessive clinical syndrome, such as Nijmegen breakage syndrome (NBS), which is characterized by spontaneous chromosomal instability carry a homozygous mutation in the NBN (NBS1) gene. The product of the gene is a part of the BRCA1-associated genome surveillance complex, which is responsible for DNA damage repair. A 5-bp deletion in exon 6 of *NBS1* (657del5) is a founder mutation present in the majority of NBS patients from Eastern Europe. The 657del5 allele (in heterozygous state) is present with a frequency of 0,6% in the Polish population. Recent studies suggested that heterozygous carriers of the 657del5 truncating mutation exhibit increased susceptibility to prostate and breast cancer, but missense variants of NBS1 have not been studied in this regard.

In order to investigate whether missense mutations of NBS1 predispose to prostate cancer and breast cancer, we assayed for the presence of three missense variants of NBS1 (Ile171Val, Arg215Trp, Glu185Gln) in 5097 men with prostate cancer, 1090 women with breast cancer and 4208 cancer-free controls using TaqMan-PCR. Genotyping call rate exceeded 98% for all variants in both cases and controls. We saw a higher frequency of Arg215Trp missense mutation in women with unselected breast cancer (0.6%) than in controls (0.6% vs 0.3%; OR = 2.5, p = 0.1). Also, the Arg215Trp variant was more frequent in women with familial breast cancer than in controls (0.8% vs 0.3%; OR=3.1, p = 0.3). The frequencies of Ile171Val, Arg215Trp and Glu185Gln variants were similar in prostate cancer cases and controls (OR between 0.9 and 1.1). The frequencies of Ile171Val and Glu185Gln variants were almost identical in breast cancer cases and controls (OR = 1.0).

This is the first large study to investigate the role of three missense mutations of NBS1 in genetic susceptibility to both prostate and breast cancer. Our results suggest that Arg215Trp missense mutation of NBS1 may be associated with two to three fold increased risk of breast cancer, but further studies are needed in this regard.

## A8 Pharmacogenetic models of adverse reactions to FAC chemotherapy in breast cancer patients

### Tęcza K, Pamuła-Piłat J, Grzybowska E

#### Center for Translational Research and Molecular Biology of Cancer, Maria Sklodowska-Curie Memorial Cancer Center and Institute of Oncology, Gliwice Branch, Poland

The differences in patients’ response to the same medication are one of the major problems in breast cancer treatment. Chemotherapy’s toxicities make a significant clinical problem due to decreased quality of life, prolongation of treatment and reinforcement of negative emotions associated with therapy.

In this study we evaluated the genetic and clinical risk factors of FAC chemotherapy-related toxicities in the group of 324 breast cancer patients. Selected genes and their polymorphisms were involved in FAC drugs transport (ABCB1, ABCC2, ABCG2, SLC22A16), metabolism (ALDH3A1, CBR1, CYP1B1, CYP2C19, DPYD, GSTM1, GSTP1, GSTT1, MTHFR, TYMS), DNA damage recognition, repair and cell cycle control (ATM, ERCC1, ERCC2, TP53, XRCC1).

The multifactorial risk models were constructed for 12 toxic symptoms, that combine genetic risk modifiers and clinical characteristics. The majority of toxicities was dependent on the modifications in components of more than one pathway of FAC drugs, while the impact level of clinical factors was comparable to the genetic ones. Furthermore, for the carriers of multiple high risk factors the chance of developing given symptom was significantly elevated. Our results therefore emphasize the complex nature of adverse effects during FAC breast cancer therapy, including the interplay among the polygenic inheritance and clinical risk factors.

It is hoped, that predictive models that engage multiple factors could be potentially useful in future personalized approach to cancer treatment. The tool that enable the separation of patients group in terms of expected toxicity and therefore allows the tailoring of treatment to the characteristics of given patients, could significantly improve its tolerance, patients’ quality of life and also outcome.

Acknowledgements

This work was financially supported by National Science Centre grant no. 2016/21/D/NZ5/01913 and grant no 2012/07/N/NZ5/00026.

## A9 3’utr polymorphisms of the XMETS genes and its potential effects on the FAC chemotherapy in breast cancer patients

### Pamuła-Piłat J, Tęcza K, Mazur M, Grzybowska E

#### Center for Translational Research and Molecular Biology of Cancer, Maria Sklodowska-Curie Memorial Cancer Center and Institute of Oncology, Gliwice Branch, Poland

Xenobiotic metabolizing enzymes and transporters (XMETs) are involved in biotransformation, detoxification and transport of therapeutic drugs. Genetic variations in the XMETs genes may modulate an activity of anticancer drugs. Single nucleotide polymorphisms (SNPs) in the regulatory sequences of genes (3’UTR) may be the cause of the individual variation in treatment response in breast cancer patients and could lead to drug-resistance/drug sensitivity.

We examined the eleven 3’UTR SNPs in nine genes in 324 breast cancer patients treated with FAC first-line chemotherapy (FAC regime combines 5-fluorouracil, doxorubicin and cyclophosphamide). In this study we analyzed genes encoding proteins involved in FAC drugs transport (ABCA1, ABCC4, ABCC1), metabolism (CYP1A2, CYP2E1, GSTM3, TYMS) and drug-induced damage repair (ERCC1, ERCC4). Only functional polymorphisms in 3’UTRs of genes were selected for the analysis.

The impact of polymorphism on therapeutic toxicity was based on multivariate models based on 12 symptoms of toxicity. Preliminary results showed the effect of 3’UTR polymorphisms of the ABCC1 and ERCC1 genes on hematological therapeutic toxicity. The preliminary results of this study confirm the value of the previously developed by our team multifactorial model of therapeutic response predictions. The potential application of the results is seen in the choice of optimal treatment regimen for the patient and in improving the quality of life of patients during 5-fluorouracil, doxorubicin and cyclophosphamide breast cancer treatment.

Acknowledgements

This work was financially supported by National Science Centre grant no. 2016/21/D/NZ5/01913 and grant no 2012/07/N/NZ5/00026

## A10 Treatment decision support for cancer - value of genetic testing

### Griesbach S

#### CeGaT GmbH, Tübingen, Germany

The underlying case for each tumor are mutations. Mutations determine whether a tumor is cancerous or benign and an increasing number of treatment options are specifically designed to target tumors with certain genetic changes. In order to decide on the optimal treatment pathway, the tumour associated genetic variants should be determined and discussed in an interdisciplinary tumor board together with all other diagnostics (e.g. CT or PET scans). Today, whole gene sequencing, performed using next generation sequencing (NGS), is the best option to determine the specific somatic mutations present in a tumor. This enables the analysis of many genes in parallel from various sources, including tumor biopsies, cell-free DNA (cfDNA), or circulating tumor cells. We highlight here an ultra-deep sequencing approach applicable for nearly every tumor type, and its use in informing treatment decisions as an integral part of a personalized therapy approach.

## A11 Similar 10-year survival in breast cancer with patients common BRCA1 mutations in Poland and Lithuania

### Elsakov P^1^, Ostapenko V², Luksyte A², Smailyte G²

#### ^1^Institute of Oncology, Vilnius University, Vilnius, Lithuania; ^1^Department of Breast Surgery and Oncology, Institute of Oncology, Vilnius University, Vilnius, Lithuania

The aim of this study was to estimate the Lithuanian population 10-year survival of a common, with differing prevalence, BRCA1 (5382insC) founder mutation associated breast cancer patients for comparison with 10-year survival rates in Poland. The study focused on 263 breast cancer patients (47 patients with strong hereditary cancer criteria and 216 patients unselected for age or family history) diagnosed in stage (TNM) I-III and treated at Vilnius Oncology Institute between 1996 and 2009. The results of our study suggested that the 10-year survival rate among breast cancer patients with BRCA1 founder mutations is similar to patients without a BRCA1 mutation (P = 0.7138). The adjusted HR associated with carrying a BRCA1 founder mutation was 1.04 (95% CI, 0.4 to 2.23; P > 0.05). The conclusions of our study are that 10-year survival rates for breast cancer patients are similar in both Poland and Lithuania, and are not dependent on their BRCA1 common founder mutation contribution or carrier status.

## A12 Quality of life of BRCA1 carriers after preventive adnexectomy

### Sawicka J^1^, Jasiewicz A^2^, Gronwald J^3^

#### ^1^Department of Oncology, Oncology Hospital, Brzozów, Poland; ^2^Diagnostic Laboratory, Oncology Hospital, Brzozów, Poland; ^3^International Hereditary Cancer Center, Department of Genetics and Pathology, Pomeranian Medical University, Szczecin, Poland

Females with mutations of BRCA1 gene have a very high risk of developing breast cancer and/or ovarian cancer. Preventive surgery (oophorectomy and hysterectomy) is a gold standard for prophylaxis for these patients, however it may result in a lower quality of life. In order to determine the impact of such surgery on the quality of life among the BRCA1 gene mutation carriers, a survey was conducted in a group of 156 women: 78 who underwent prophylactic surgery, and 78 women who did not undergo any prophylactic surgery (a self-prepared survey, WHOQOL-BREF short version consisting of 26 questions questionnaire, and the LISAT 11 questionnaire were used).

**Results and Conclusions:**

(I) The social status and level of education have an impact on the incidence of undergoing preventive surgery among BRCA1 gene mutation carriers; (II) Undergoing preventive gynecological surgery by BRCA1 mutation carriers, significantly increases the sense of security regarding the risk of developing breast and ovarian cancer; (III) Preventive surgery among BRCA1 gene mutation carriers increases incidence of menopausal symptoms, and this applies both to women using and not using HRT; (IV) BRCA1 mutation carriers who underwent preventive surgery, are strongly convinced of prophylactic surgery relevance; (V) Undergoing preventive surgery does not adversely affect the physical, psychological, environmental and social factors of quality of life among the BRCA1 gene mutation carriers.

## A13 The role of zinc concentration and alterations in genes associated with zinc metabolism in cancer- retrospective studies

### Kaczmarek K^1^, Marciniak W^2^, Muszyńska M^2^, Baszuk P^1^, Sukiennicki G^1^, Lener M^1^, Durda K^1^, Jaworska-Bieniek K^1^, Gromowski T^1^, Prajzendanc K^1^, Łukomska A^1^, Huzarski T^1^, Gronwald J^1^, Cybulski C^1^, Dębniak T^1^, Morawski A^2^, Jakubowska A^1^, Lubiński J^1,2^

#### ^1^Department of Genetics and Pathology, International Hereditary Cancer Center, Pomeranian Medical University, Szczecin, Poland; ^2^Read – Gene, S.A., Grzepnica, Poland

**Introduction**

Recently, it has been reported that zinc may play role in chemoprevention, and its level may be associated with occurrence of cancers. In the present study we evaluated the association between serum zinc level and polymorphisms in zinc-dependent genes with cancer incidence in Poland.

**Methods**

The study group consisted of 845 individuals affected with cancer (prostate, breast, lung, colon) and 845 healthy controls. Serum zinc level was measured by ICP-MS and genotyping of 5 single nucleotide polymorphism in 5 genes (*MMP-1, MMP-2, MMP-7, MMP-13, MT2A*) was performed.

**Results**

The mean serum zinc level was higher in prostate cancer patients comparing to healthy controls. Increased Zn level may be associated with higher probability of diagnosing prostate cancer in Poland. Zn level was not associated with probability of diagnosing lung, colon and breast cancer. Among five analyzed genetic variations one polymorphism rs11568818 in *MMP-7* showed a correlation with prostate cancer risk. None of the tested SNPs in *MMP-1, MMP-2, MMP-7, MMP-13, MT2A* was associated with Lu, Col, Br cancer risk in Poland.

**Conclusions**

Results from this study suggests that serum zinc level may be associated with cancer incidence. In Poland polymorphism rs11568818 in *MMP-7* was associated with prostate cancer risk.

Acknowledgments

This project was financially supported by National Science Centre, grant no. 2012/07/N/NZ4/02433.

## A14 The role of iron concentration and alterations in genes associated with iron metabolism in cancer

### Sukiennicki G^1^, Baszuk P^1^, Marciniak W², Muszyńska M², Jaworska – Bieniek K^1^, Kaczmarek K^1^, Prajzendanc K^1^, Łukomska A¹, Lener M¹, Lubiński J¹^,^², Jakubowska A¹

#### ^1^Department of Genetics and Pathology, Pomeranian Medical University, Szczecin, Poland; ^2^Read - Gene S.A., Szczecin, Poland

**Introduction**

Iron plays an important role in many metabolic processes, is included in the delivery of oxygen to cells and redox processes. Deficiency and excess of iron can lead to multiple organ failures. The latest studies show that the iron can significantly influence the risk of cancer development and progression. Scientists reported association between high serum iron level and the risk of colon, liver, stomach and breast cancers. The low iron level was detected in patients with colorectal, breast, bladder and lung cancers. However, there are also studies in which authors did not find any correlation between iron levels and the risk of cancer. The discrepancies between studies might be explained in part by the influence of other factors e.g. genetic variants in proteins involved in iron metabolism and redox processes.

**Aim**

The aim of the study was to analyze correlation between of the serum Fe level, Fe parameters and variations in genes coding proteins involved in iron metabolism with occurrence of lung, prostate, colorectal and breast cancers.

**Materials and methods**

The study group consisted of 850 cancer patients, including breast (300), lung (200), prostate (200) and colon (150), and equal number of healthy controls matched to cases by sex, year of birth (+/- 3 years), cancer family history among I° relatives, smoking (+/- 10%) and adnexectomy status in group with breast cancer.

From all individuals were collected serum samples and analyzed iron concentration and iron metabolism parameters (UIBC, concentration and transferrin saturation and serum ferritin). Serum samples from cancer patients were collected at the time of cancer diagnosis, before treatment. In addition, seven variants in seven genes (rs1799945 in HFE, rs3817672 in TFR1, rs10421768 in HAMP, rs1049296 in TF, rs4880 in SOD2, rs1001179 in CAT, rs1050450 in GPX1) associated with iron metabolism and redox processes were genotyped.

**Results**

Results have shown an association of Fe level with lung, prostate and colon cancers. Fe level >1497μg/l and ferritin >301.27 were significantly associated with >2 -fold higher probability of lung cancer diagnosis, whereas Fe concentration in range 1113.51-1471.76μg/l was associated with >2-fold lower probability of prostate cancer diagnosis. In group of colon cancer probability of cancer diagnosis was significantly decreasing with increased Fe level >637.78μg/l.

Analysis of genotypes revealed and association of TT genotype in rs1049296 in TF gene with prostate cancer risk and GG genotype in rs10421768 in HAMP with lung cancer risk.

**Conclusions**

Results suggest that Fe level potentially may be a diagnostic marker for lung, prostate and colon cancers. Variation in TF and HAMP genes may be significantly associated with prostate cancer and lung cancer risk, respectively.

This project was financially supported by National Science Centre, grant no. 2013/11/N/NZ4/02250.

## A15 Blood cadmium (Cd) level as a marker of cancer risk in BRCA1 (+) prospective cohort study

### Marciniak W^1,2^, Derkacz R^1,2^, Baszuk P^1,2^, Kuświk M^2^, Kaczmarek K^2^, Muszyńska M^1^, Lener M^2^, Huzarski T^1,2^, Gronwald J^1,2^, Oszurek O^2^, Tołoczko-Grabarek A^2^, Cybulski C^1,2^, Dębniak T^2^, Morawski A^1^, Jakubowska A^1,2^, Lubiński J^1,2^

#### ^1^Read - Gene S.A., Szczecin, Poland; ^2^Department of Genetics and Pathology, Pomeranian Medical University, Szczecin, Poland

Cadmium is recognized by IARC as a 1st class human carcinogen. It is widely spread in the environemnt. Major sources of cadmium exposure are occupation, water, food and cigarettes. Mechanisms of carcinogenesis induced by this microelement remains still unclear. There are few posible ways such as direct interaction with DNA strand, generation of free radicals (ROS) and interaction with crucial enzymes. According to latest papers cadmium level in biological media is linked to occurence of lung, breast, kidney, bladder, pancreas and prostate cancers in human.

We investigated the relations between cadmium level in blood and cancer incidence in Polish BRCA1 founder mutation carriers. The study was performed on 455 subjects with mean follow-up period of 25 months.

Our prospective cohort study reveals that low cadmium level is associated with lower risk of cancer in BRCA1 carrier subpopulation of Polish women. Our proposed threshold for safe cadmium level is set at 0,32 μg/l. Results for this threshold gives us 5 times lower risk for womans with blood cadmium level below 0,32 μg/l comparing with those above that value [OR=0,1994; p=0,016; 95%CI: 0,04684-0,8487]. That correlation is extremely strong especially in younger woman, under 51 years old. In woman, under 51 years old, carriing any of founder mutations in BRCA1 etimated cancer risk for cadmium level below 0,32 μg/l is nearly 10 times lower than for those with higher cadmium levels [OR=0,1136; p=0,007; 95%CI: 0,0151-0,8553].

This study shows an importance of cadmium level monitoring in blood of young BRCA1 carriers. Our results need to be confirmed by further investigations.

## A16 Arsenic as a marker of cancer risk

### Derkacz R^1,2^, Marciniak W^1,2^, Baszuk P^1,2^, Kuświk M^2^, Kaczmarek K^2^, Muszyńska M^1^, Lener M^2^, Huzarski T^1,2^, Gronwald J^1,2^, Oszurek O^2^, Tołoczko-Grabarek A^2^, Cybulski C^1,2^, Dębniak T^2^, Morawski A^1^, Jakubowska A^1,2^, Lubiński J^1,2^

#### ^1^Read - Gene S.A., Szczecin, Poland; ^2^Department of Genetics and Pathology, Pomeranian Medical University, Szczecin, Poland

**Introduction**

Arsenic is considered by International Agency for Research of Cancer as human carcinogen (Group 1) [1]. Considered as a risk factor in kidney [2], bladder [3], skin [4] and lung cancer [3]. Futhermore, there is an inverse correlation between a risk of bladder cancer and arsenic. Arsenic range in drinking water 3.0-6.0 μg/L decreased the risk of bladder cancer [5].

Arsenic is element which occurs naturally in the environment. Analysis of arsenic level is obligatory for determining food and drugs impurities. Sources of this element are: volcanic eruption, burning coal, ground water and food, especially seafood and rice.

**Methods**

The prospecitve cohoty of women BRCA1 consisted od 32 individuals with new cancer incidents identified during the follow-up and 416 uneffected controls with no cancer incident at follow-up.

The men’s prospecitve study include 28 cases with cancer incidents identified during the follow-up and 56 uneffected men with no cancer incident at baseline or follow-up. Cases and controls paired 1:2 by year of birth (+/- 3 years), smoking status (pack-year), family history of cancer (number of affected first-degree relatives. Supplements users were excluded.

Arsenic blood level was measured by ICP-MS Elan DRC-e (PerkinElmer).

**Results**

The results of women’s BRCA1 carrier before used oral contraceptives more than 1 year was unexpected. High arsenic blood level (>1.3 μg/L) decresce risk of cancer over 11 times (p=0.0029, OR=11.977; 95%CI: 1.563-91.75. The results of men’s under 60 years old are also unexpected. High arsenic blood level (>1.0 μg/L) decrease the risk of cancer over 12 times (p=0.0007, OR=12.650; 95%CI: 2.826-56.618).

**Conclusions**

Results from this study suggest that arsenic blood level can be a good marker of cancer risk among women BRCA1 carries and men in Poland.

References

[1] http://monographs.iarc.fr/ENG/Classification/latest_classif.php

[2] Hopenhayn-Rich C., Biggs M.L., Fuchs A., Begoglio R., Tello E.E., Nicolli H., Smith A.H.: Bladder cancer mortality associated with arsenic in drinking water in argentina. *Epidemiology* (1996); 7(2), s. 117 – 124.

[3] Mostafa M.G., McDOnald J.C., Cherry N.M.: Lung cancer and exposure to arsenic in rural Bangladesh. *Occup. Environ. Med.* (2008); 65(11), s. 765 - 768.

[4] Karagas M.R., Stukel T.A., Morris J.S., Tosteson T.D., Wei J.E., Spencer S.K., Greenberg E.R.: Skin cancer risk in relation to toenail arsenic concetration in a US population-based case-control study. *Am. J. Epidemiol.* (2001); 153(6), s. 559 – 565.

[5] Lamm S.H., Engel A., Kruse M.B., Feinleib M., Byrd D.M., Lai S., Wilson R.: Arsenic in drinking water and bladder cancer mortality in the United States: an analysis based on 133 U.S. counties and 30 years of observation. *J. Occup. Environ. Med.* (2004); 46, s. 298 – 306.

## A17 Clinical characteristics of colorectal cancer in patients diagnosed with CHEK2 and NOD2 gene mutations

### Wiśniowski R^1^, Kurzawski G^2^, Suchy J^2^, Cybulski C^2^, Wokołorczyk D^2^, Byrski T^3^, Al-Amawi T^4^, Kładny J^4^, Lubiński J^2^

#### ^1^Beskid Oncology Center – John Paul II Municipal Hospital in Bielsko-Biała, Bielsko-Biała, Poland; ²Department of Genetics and Pathology Pomeranian Medical University in Szczecin, Szczecin, Poland; ^3^Department of Oncology and Chemotherapy Pomeranian Medical University in Szczecin, Szczecin, Poland; ^4^Department of General and Oncological Surgery Pomeranian Medical University in Szczecin, Szczecin, Poland

**Introduction**

Malignant neoplasms of the colon and rectum are among the most common cancers in humans. They are largely related to genetic predisposition. Some of the best-described genes for multi-organ predisposition to cancer are the CHEK2 and NOD2 genes. The purpose of this study was to validate existing clinical characteristics of colorectal carcinoma in CHEK2 and / or NOD2 mutation carriers, and especially 5-year survival rate.

**Material and methods**

The methodology of work was based on molecular and clinical analysis of 852 patients sequentially diagnosed for colorectal and rectum cancer treated in the Beskid Oncology Center in Bielsko Biała and Independent Public Clinical Hospital No. 2 PUM in Szczecin. The study group was analyzed for their clinical stage, the age when the disease started, 5-year survival rate and the presence of the CHEK2 mutation.

**Results**

Complete data was obtained from 553 people. 34 mutations in the CHEK2 gene and 57 mutations in the NOD2 gene were detected. There was no statistically significant trend for younger age at which the disease started, or a higher degree of clinical progression in patients with colorectal cancer and CHEK2 or NOD2 gene mutation. The largest difference in the risk of death between the patients at I and II stage, and the patients at III and IV stage of clinical progression was observed in CHEK 2 (OR-1.8, p-0.39) mutation carriers.

## A18 A retrospective evaluation of 512 results of UroVysion test applications

### Kałużewski T^1^, Morzuch L^1^, Jędrzejczyk A^2^, Marks P^2^, Różniecki M^3^, Kałużewski B^1^

#### ^1^GENOS Laboratory of Medical Genetics, Zapolice, Poland; ^2^Department of Urology, the JP II Voivodeship Hospital, Belchatow, Poland; ^3^Non-Public Department of Urology Rozniecki and Partners, Voivodeship Hospital, Zduńska Wola, Poland

In the process of carcinogenesis, there is a time point of cellular genome destabilisation, a frequentsymptom of which is chromosomal aneuploidy of chromosomes 3, 7 and 17 and a loss of locus 9p21(UroVysion Test). In the course of 5 years, we carried out 512 UroVysion tests; in 68%, the patientswere referred for diagnostics of urinary bladder cancer, in 8.4% for therapeutic effect follow up, in 2%it was dysuria, in 2.7% haematuria, in 0.5% chronic, recurrent cystitis, other indications - 1.4%, noprecise indications - 13.1%. Diagnostic criteria: 25 morphologically abnormal cells were evaluated. Aresult was regarded positive when more than 10 cells demonstrated a loss of 9p21 fragment (thepresence of one or the loss of both signals) or if more than 4 cells showed polysomy of twochromosomes (among chromosomes 3,7,17) or if more than 10 cells indicated polysomy for one of the above-mentioned chromosomes. Positive results were obtained in 56% of the cases. In the group with negative results, our attention was drawn to a statistically higher percent of rearrangements in 9p21 region, compared to changes in chromosomes 3, 7, 17. That observation did confirm the known factthat the change in question is the earliest one in the process of carcinogenesis. What was a surprising new observation was that the change was found in the group with negative results.

## A19 Frequency of BRCA1 and BRCA2 founder mutations among ovarian cancer patients from Podkarpackie Voivodeship

### Kluz T^1^, Jasiewicz A^2^, Gronwald J^3^

#### ^1^Department of Gynecology and Obstetrics, Chopin Hospital of Rzeszow, Rzeszow, Poland; ^2^Diagnostic Laboratory, Oncology Hospital, Brzozów, Poland; ^3^International Hereditary Cancer Center, Department of Genetics and Pathology, Pomeranian Medical University, Szczecin, Poland

It was shown that about 12-13% of ovarian cancers in Polish population are caused by founder mutations in BRCA1 gene. Some regional differences were reported. In our study we evaluated the frequency of BRCA1 and BRCA2 founder mutations among ovarian cancer patients from Podkarpackie Voivodeship. DNA testing for 8 BRCA1 and 5 BRCA2 founder mutations was performed in 160 consecutive patients diagnosed with ovarian cancer in Rzeszów Regional Hospital in 2013-2016. BRCA1 mutation was diagnosed in 10 (6,25%) patients. BRCA1 mutations - 5382 ins C was observed in 6 patients; ex. 5 300 T G in 3 patients; and 794delT I 1 patient. No BRCA2 mutation was diagnosed. The frequency as well as mutation spectrum in some extend are different from general population. BRCA1/2 NGS should be performed in larger number of patients.

## A20 Constitutional methylation of BRCA1 gene in breast cancer

### Prajzendanc K^1^, Kaczmarek K^1^, Łukomska A^1^, Sukiennicki G^1^, Szwiec M^2,3^, Huzarski T^1^, Wojdacz TK^4^, Lubiński J^1^, Jakubowska A^1^

#### ^1^Department of Genetics and Pathology, International Hereditary Cancer Center, Pomeranian Medical University, Szczecin, Poland; ^2^Holy Cross Cancer Center, Counselling Unit, Kielce, Poland; ^3^Regional Oncology Center, Counselling Unit, Opole, Poland; ^4^Aarhus Institute of Advanced Studies, University of Aarhus, Aarhus, Denmark

Methylation of CpG islands in DNA is an epigenetic modification that causes silencing of genes and might be associated with cancer risk if present in peripheral blood. It has been shown that constitutional methylation of *BRCA1* promoter correlates with breast cancer risk, especially with triple-negative tumours. In this study we evaluated breast cancer risk depending on methylation of *BRCA1* promoter in peripheral blood. We examined three groups of women: 519 unselected breast cancer cases, 500 triple-negative breast cancer cases and 500 healthy controls. All women were negative for *BRCA1* germline mutations. Moreover, we tested 161 FFPE tumour tissues from our cases. Methylation in all samples was measured using methylation-sensitive high-resolution melting (MS-HRM). Samples with any detectable methylation level were considered as positive. The results show that *BRCA1* methylation may be associated with risk of triple-negative breast cancer and correlates with methylation in paired tumours. Obtained results confirm previous studies, in particular our pilot study.

The study was financially supported by the National Science Centre (NCN) grant 2014/15/B/NZ1/03386.

## A21 CD36 – A plausible modifier of disease phenotype in familial adenomatous polyposis

### Holmes M^1^*, Connor T^1,2^*, Singh AK^3^, Oldmeadow C^4^. Pockney PG^1^, Talseth-Palmer BA^5,6,7^, Scott RJ^2,5^

#### ^1^School of Medicine and Public Health, Faculty of Health and Medicine, University of Newcastle, Newcastle, NSW, Australia; ^2^Pathology North, NSW Health Pathology, Newcastle, Australia; ^3^Department for Medical Genetics, St. Olavs Hospital, Trondheim, Norway; ^4^Centre for Clinical Epidemiology and Biostatistics, University of Newcastle, Newcastle, NSW, Australia; ^5^School of Biomedical Science and Pharmacy, Faculty of Health and Medicine, University of Newcastle and Hunter Medical Research Institute, Newcastle, Australia; ^6^Department of Clinical and Molecular Medicine, Faculty of Medicine and Health Sciences, Norwegian University of Science and Technology, Trondheim, Norway; ^7^Clinic for Medicine, Møre og Romsdal Hospital Trust, Molde, Norway

*These authors contributed equally to this manuscript

Familial Adenomatous Polyposis (FAP) is a well characterised genetic predisposition to early onset colorectal cancer (CRC) that is preceded by polyposis of the colon and rectum. Multiple studies have previously described a genotype/phenotype correlation between causative variants in *APC* and disease severity. Whilst this association has been well established, there is evidence that modifier genes may influence the disease phenotype. Animal models have consistently suggested the role of modifier genes in determining disease phenotype, yet none have been substantiated in the human population.

In this study we have investigated three variants within *CD36* in 275 FAP patients all of whom harboured causative variants in *APC*. Three single nucleotide polymorphisms (SNPs) in *CD36*; rs1049673, rs1761667 and rs1984112 were studied to determine if one or more of them were associated with disease expression in FAP patients. The results revealed an increased risk of polyposis in patients harbouring the wildtype genotype of rs1761667 but most interestingly a substantially low age of polyposis diagnosis for patients belonging to the severe FAP group (harbouring variants in mutation cluster region (MCR)) for SNPs rs1761667 and rs1984112.

This study provides evidence for patients belonging to the MCR FAP group harbouring specific genotypes for two SNPs in *CD36* to initiate screening/treatment for FAP at much earlier ages than other FAP groups (AFAP and classic FAP). The findings need to be verified in an independent FAP patient cohort.

